# Radiation safety issues with positron‐emission/computed tomography simulation for stereotactic body radiation therapy

**DOI:** 10.1120/jacmp.v9i3.2763

**Published:** 2008-06-23

**Authors:** William T. Kearns, James J. Urbanic, Carnell J. Hampton, Kevin P. McMullen, A. William Blackstock, Volker W. Stieber, William H. Hinson

**Affiliations:** ^1^ Department of Radiation Oncology Wake Forest University Baptist Medical Center Winston–Salem North Carolina; ^2^ Department of Radiation Oncology Forsyth Medical Center Winston–Salem North Carolina

**Keywords:** Positron‐emission tomography, radiation safety, stereotactic body radiation therapy, SBRT, simulation

## Abstract

Stereotactic body radiation therapy (SBRT) simulations using a Stereotactic Body Frame (SBF: Elekta, Stockholm, Sweden) were expanded to include ^18^F‐deoxyglucosone positron‐emission tomography (FDG PET) for treatment planning. Because of the length of time that staff members are in close proximity to the patient, concerns arose over the radiation safety issues associated with these simulations. The present study examines the radiation exposures of the staff performing SBRT simulations, and provides some guidance on limiting staff exposure during these simulations. Fifteen patients were simulated with PET/CT using the SBF. Patients were immobilized in the SBF before the FDG was administered. The patients were removed from the frame, injected with FDG, and allowed to uptake for approximately 45 minutes. After uptake, the patients were repositioned in the SBF. During the repositioning, exposure rates were recorded at the patient's surface, at the SBF surface, and at 15 cm, 30 cm, and 1 m from the SBF. Administered dose and the approximate time spent on patient repositioning were also recorded. The estimated dose to staff was compared with the dose to staff performing conventional diagnostic PET studies. The average length of time spent in close proximity (<50 cm) to the patient after injection was 11.7 minutes, or more than twice the length of time reported for diagnostic PET staff. That time yielded an estimated average dose to the staff of 26.5*m*Sv per simulation. The annual occupational exposure limit is 50 mSv. Based on dose per simulation, staff would have to perform nearly 1900 SBRT simulations annually to exceed the occupational limit. Therefore, at the current rate of 50–100 simulations annually, the addition of PET studies to SBRT simulations is safe for our staff. However, ALARA (“as low as reasonably achievable”) principles still require some radiation safety considerations during SBRT simulations. The PET/CT‐based SBRT simulations are safe and important for treatment planning that optimizes biologic dose distribution with highly accurate and reproducible target definition.

PACS numbers: 87.57.uk, 87.59.bd

## I. INTRODUCTION

Stereotactic body radiation therapy (SBRT) is emerging as a viable option for the treatment of tumors in the torso and abdomen, particularly those of the lung and liver.^1^, ^9^ In SBRT treatments, the patient is immobilized, and a stereotactic coordinate system is used in targeting the treatment volume. Because SBRT allows for more precise localization of the treatment target in the stereotactic coordinate system, treatment plans can in turn be more conformal. Several studies have been published describing the various approaches to SBRT treatments in various extracranial sites.(10–13)

Because of the high level of conformality required for SBRT treatments, target definition becomes more critical in treatment planning. Several groups have published findings outlining a growing interest in the use of positron‐emission tomography (PET) images for target definition in non‐small‐cell lung cancer.^14^, ^17^ Studies in other tumor sites, including esophagus^(18)^ and head‐and‐neck cancers,^(19)^ have also been published. Other authors have provided some guidelines for the use of PET for treatment planning and have noted issues regarding the use of PET‐defined volumes.^(20–22)^ Despite the concerns, PET is becoming an integral part of advanced treatment planning in both conventional radiation therapy and SBRT planning.

Since 2003, our institution has used the Stereotactic Body Frame (SBF: Elekta, Norcross, GA), a commercially available stereotactic immobilization device, to perform more than 150 SBRT simulations. Since the acquisition of a dedicated PET/computed tomography (CT) simulator in 2005, we have routinely incorporated PET/CT simulation for multiple sites, including head‐and‐neck, lung, and various others, accounting, in all, for more than 150 PET/CT simulations.

For SBRT patients, simulations have often included an ${}^{18}F$‐deoxyglucose (FDG) PET scan in addition to the treatment planning CT. Before acquisition of the dedicated PET/CT simulator, we would routinely fuse externally performed PET scans to the treatment planning CT. The need for a high level of reproducibility combined with the critical additional information for target localization in SBRT planning that is provided by PET, has led us to perform PET/CT simulations with the patient in the proper treatment position in the SBF. This change in the simulation process, together with the general use of PET/CT for simulation, has prompted some concern over staff exposure rates during a PET/CT simulation. It was therefore important to examine the effect that these changes in the simulation process were having on staff radiation safety and to investigate strategies to streamline the process to minimize staff exposure. Given that our simulation time is longest for SBRT patients, we felt that this study would provide a conservative estimate for all PET/CT simulations.

The exposure rates for diagnostic PET staff are well documented.^(21,23–31)^ The findings in those studies were based on diagnostic PET scans, and the exposure estimates and measurements reported were those made in traditional radiology‐based PET centers. The studies outlined process flow strategies to limit the dose to occupational workers in a traditional PET setting. Zeff and Yester outline time intervals of staff exposures throughout the PET process, including dose preparation and administration, escorting the patient back to the scan room, aligning the patient on the table, repositioning the patient for PET, and removing the patient from the scanner room.^(26)^ For each of those time intervals, time of exposure and exposure dose to staff were reported.

Because of the similarities in the tasks, the time intervals for staff exposures during radiation oncology simulations should be very similar to those of the diagnostic PET scans, except for the time spent aligning the patient on the table and repositioning the patient for PET. Because staff exposures during dose preparation and administration are similar to those in Zeff and Yester's study, the present report focuses on the time of staff exposure during patient alignment and repositioning in the scanner room. Simulations of SBRT patients, particularly those done in a stereotactic immobilization device like the SBF, add another layer of complexity to the simulation process. They require special care in the realignment of the patient in the immobilization device for the PET image acquisition. Because of the time taken by the simulation process, we studied process flow and radiation safety issues for radiation oncology staff (physicists and therapists). Here, we report the findings and offer some guidelines for process flow modifications to limit the exposure to staff involved in these simulations.

## II. MATERIALS AND METHODS

Fifteen patients underwent SBRT simulation using PET/CT. All simulations were performed in a General Electric Discovery ST, 8‐slice PET/CT Scanner with a General Electric Advantage Workstation (GE Healthcare, Waukesha, WI). Patients were immobilized using the Elekta SBF. Exposure rates were recorded using an Innovision 451P Survey Meter (Fluke Biomedical, Everett, WA). Personal dosimetry readings were obtained using a Thermo Scientific dosimeter, model EPD mk2 (Thermo Scientific, Waltham, MA). The retrospective review of the relevant data was done with institutional review board approval.

**Figure 1:**
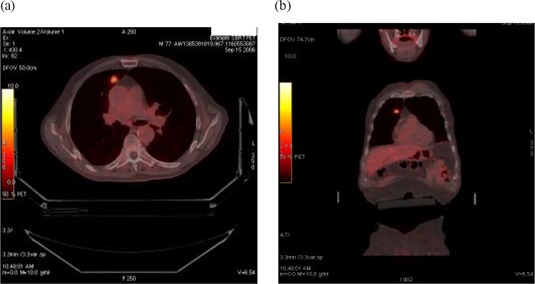
Sample positron‐emission/computed tomography image of a stereotactic body radiation therapy patient.

Before the start of the simulation, an intravenous line is placed for administration of FDG and CT contrast (if ordered). The dose is prepared by a nuclear medicine technologist in our department, as would be done in a diagnostic PET facility. Upon arrival in the simulation suite, the patient is positioned and immobilized in the SBF, according to the manufacturer's guidelines. A conventional, non‐contrasted CT scan is acquired to check for optimal patient position in the SBF. If the patient requires repositioning, it is done then, before the FDG is administered. For patients who can tolerate it, the abdominal compression paddle is used to limit motion related to respiration. The patient is then removed from the frame, injected with FDG in the uptake room, and allowed to uptake for approximately 45 minutes. Patients are asked to void before returning to the simulation room. Patients are then repositioned in the SBF typically with 1 physicist and 2 simulation staff present. During repositioning, records of instantaneous peak exposure rates were acquired adjacent to the patient's surface, the SBF surface, 15 cm from the SBF, 30 cm from the SBF, and 1 m from the SBF in free air without scattering material. For consistency, all readings were taken at the level of the umbilicus on the patient's right side. The length of time for the repositioning, from the time that the patient returned to the scan room until image acquisition started, was also recorded and rounded to the nearest minute. A PET scan was acquired, together with a contrasted (if ordered) or non‐contrasted CT scan (Fig. 1). All acquired datasets were fused with frame‐to‐frame matching and composite gross tumor volumes were contoured using the second CT scan as the base planning scan.

## III. RESULTS

For the 15 SBRT patients studied, Table 1 lists the average times spent adjacent to the patient during alignment and repositioning. The average length of staff exposure during these SBRT simulations was 11.7±3.2 minutes, with a range of 6 – 18 minutes. The average injected FDG activity was 13.7±2.1 mCi, with a range of 10.0 – 17.6 mCi. The data show that the exposure rates varied greatly at the patient's surface, but were uniform at distances greater than 30 cm. Typical working distance while actively positioning the patient was in the 15 – 30 cm range. Given an average exposure of 94±15 μSv/h at 15 cm for 10 minutes, the maximum estimated exposure would be no more than 0.02 mSv for the patient alignment and repositioning procedure, assuming that the staff member is within 15 cm of the frame the entire time, which is typically not the case. Again, these results do not include source handling and injection exposure rates.^(26)^

**Table 1:** Exposure times and rates for stereotactic body radiation therapy simulation patients

				*Exposure rate (μSv/h) at*			
*Patient*	*FDG activity (mCi)*	*Time near to patient (min)*	*patient surface*	*SBF surface*	*15 cm from surface*	*30 cm from surface*	*1 m from surface*
1	14.2	14	280	174	99	50	15
2	10.8	8	148	138	88	49	14
3	14.3	10	290	195	85	50	15
4	14.7	6	200	155	87	60	19
5	10.0	15	189	145	90	55	18
6	15.0	10	260	188	125	66	21
7	17.6	10	290	190	103	63	25
8	14.0	18	193	159	75	49	21
9	13.4	14	260	198	113	54	17
10	13.1	9	310	144	82	56	18
11	12.0	13	280	153	73	45	21
12	14.9	12	310	200	112	61	29
13	14.8	10	290	172	102	72	27
14	15.4	12	350	192	102	68	28
15	10.6	15	250	141	78	47	20
**Average**	13.7±2.1	11.7±3.2	260±55	170±23	94±15	56±8	21±5

$\text{FDG}={\;}^{18}\text{F‐deoxyglucose}$; SBF=stereotactic body frame.

To verify the cumulative dose to staff during patient alignment and repositioning, a therapist or a physicist was equipped with a personal dosimeter while working with 5 of the 15 patients. The cumulative dose readings for these 5 patients were 8.4, 8.8, 7.0, 8.0, and 9.0 μSv, for an average dose equivalent of 8.2 μSv. Again, these doses were measured only for the patient alignment and repositioning phase of the PET study.

## IV. DISCUSSION

The current exposure limit for occupational workers is 50 mSv annually. Zeff and Yester reported a cumulative dose range of 7.8 – 11.2 μSv/study for a diagnostic PET technologist. They reported that, at the time of their study, their institution averaged 6 PET patients daily. Assuming the maximum exposure for each of 6 patients daily for 5 days each week for 52 weeks per year, the technologists would still receive only 17.5 mSv annually, well below the limit of 50 mSv annually. In their data, Zeff and Yester reported that 1.7 μSv and 0.9 μSv are delivered during the patient alignment and repositioning, for a total of 2.6 μSv. Therefore, for the worst case, the technologists would receive 8.6 μSv during dose preparation and administration. Chiesa et al. reported whole‐body doses to staff of 5.9 μSv per patient.^(30)^ Similarly, Benatar et al.^(27)^ measured 5.5 μSv per patient and 14.4 μSv daily for their 2–3 daily patients.

Our dose preparation and administration process is similar to that for a diagnostic PET process, including use of appropriate syringe shields and nuclear medicine processes, so that the dose to our therapists should be very similar to the doses previously reported for these tasks.

The dose to our personnel during the patient alignment and repositioning phases is the focus of the present study. Assuming that the therapists are, on average, 15 cm from the SBF during the alignment process, the estimated doses during that time would be

(1) $$Estimated\;dose=(94\mu Sv/hr)(11.4\min)\left(\frac{1hr}{60\min}\right)=17.9\mu Sv.$$

That estimate is much larger than the 2.6 *m*Sv that Zeff and Yester report, as would be expected for the increased exposure time during SBRT patient alignment and repositioning. Adding the additional dose (8.6 *m*SV, worst case from Zeff) delivered during dose preparation and administration, our PET technologists could receive 26.5 *m*Sv per PET study. Given that average dose, our personnel would have to simulate nearly 1900 SBRT patients annually to exceed the occupational limit of 50 mSv. Our current patient load is far below that limit, and so we feel that staff exposure for this type of simulation is acceptable.

The actual absorbed dose was much less than the estimated dose. A personal dosimeter was used to measure actual dose during simulation of 5 of the 15 patients. These measurements revealed that, on average, staff received 8.2 μSv during patient alignment and repositioning, or about one half the dose estimated earlier. That difference is the result of staff not being within 15 cm of the SBF for the entire repositioning process. The absorbed dose readings further confirm that these PET simulations are safe for personnel.

Although the SBRT simulations are “safe” and well under occupational limits, ALARA (“as low as reasonably achievable”) principles still require an examination of the SBRT simulation process so as to limit the dose to staff as low as possible.^(32)^ In an effort to lower staff exposure, we designed a process to limit the time that staff are in close proximity to the patient after dose administration. The process begins with proper patient selection. The physical requirements for treatment in the SBF go beyond simply fitting into the frame. Patients must be able to maneuver themselves into the frame and keep their arms above their head for the duration of the imaging study. Patients that can readily perform these tasks are much easier to realign in the frame after uptake. Preliminary preparation of the patient in the SBF can take more than 20 minutes. By contrast, Zeff reports one diagnostic case of a very sick patient that required staff to reposition the patient several times, yielding a time of more than 5 minutes with the staff in close proximity to the patient. By performing that step before FDG injection, staff avoid unnecessary exposure. However, a significant drawback is the possible jeopardy to the timing of the FDG injection. Given the FDG half life of approximately 109.8 minutes, the target activity can be dramatically reduced should any delays occur in the process. For example, a target dose of 15 mCi can become 12.4 mCi with just a 30‐minute delay or 10.2 mCi with a 60‐minute delay. Such an event happened with one of our patients who experienced problems with both intravenous access and positioning. Given her low body weight, the physician decided to proceed with the study even though the injected activity was just 10.8 mCi.

During the simulation, personnel are instructed to recognize that the patient is a radioactive source and, when interacting with the patient, to follow guidelines identical to those for limiting exposure to sealed sources, acknowledging that the patient's safety and well‐being remains the utmost priority. If at any time during the simulation a question arises that requires some delay, staff are instructed to stand away from the patient while waiting for the answer. It should be noted that at a meter, all patients, regardless of injected activity and body weight, yielded an exposure of less than 30 μSv/h. Our personnel have been instructed to maintain position beyond that distance or to remain in the shielded console area unless actively working with the patient. Similar guidelines exist for all of our PET/CT simulation patients with regard to workflow and ALARA principles.

## IV. CONCLUSION

Body frame simulation with PET/CT is feasible and does not add a substantial burden of time to the simulation process. Radiation safety issues are minimized by using appropriate ALARA principles. We plan to use this PET/CT‐based simulation process for treatment planning that optimizes biologic dose distribution with highly accurate and reproducible target definition.
